# Barcoding of Italian mosquitoes (BITMO): generation and validation of DNA barcoding reference libraries for native and alien species of Culicidae

**DOI:** 10.1186/s13071-024-06478-0

**Published:** 2024-09-28

**Authors:** Beatrice Bisaglia, Michele Castelli, Laura Soresinetti, Agata Negri, Irene Arnoldi, Fabrizio Montarsi, Federica Gobbo, Francesco Defilippo, Emanuele Callegari, Marco Di Luca, Mattia Calzolari, Valentina Mastrantonio, Daniele Porretta, Gentile Francesco Ficetola, Davide Sassera, Paolo Gabrieli, Claudio Bandi, Sara Epis

**Affiliations:** 1 Department of Biology and Biotechnology “Lazzaro Spallanzani”, University of Pavia, 27100 Pavia, Italy; 2 Department of Biosciences and Pediatric Clinical Research Center “Romeo Ed Enrica Invernizzi”, University of Milan, 20113 Milan, Italy; 3 Istituto Zooprofilattico Sperimentale Delle Venezie, 35020 Legnaro, Padua Italy; 4 Istituto Zooprofilattico Sperimentale Della Lombardia E Dell’Emilia-Romagna “B. Ubertini” (IZSLER), 25124 Brescia, Italy; 5 Department of Infectious Diseases, Istituto Superiore Di Sanità, 00161 Rome, Italy; 6 Department of Environmental Biology, La Sapienza” University of Rome, 00185 Rome, Italy; 7 Department of Environmental Science and Policy, University of Milan, 20113 Milan, Italy; 8 Fondazione Istituti Di Ricovero E Cura a Carattere Scientifico (IRCCS) Policlinico San Matteo, 27100 Pavia, Italy

**Keywords:** EDNA, 16S rDNA, Cytochrome* c* oxidase, Internal transcribed spacer 2, Mosquito, Biodiversity, Species identification

## Abstract

**Background:**

Mosquitoes (Culicidae), as disease vectors, represent a risk for human health worldwide. Repeated introductions of alien mosquito species and the spread of invasive species have been recorded in different countries. Traditionally, identification of mosquitoes relies on morphological observation. However, morphology-based identification is associated with a number of potential disadvantages, such as the high level of specialisation of the operator and its limited applicability to damaged samples. In these cases, species identification is achieved through molecular methods based on DNA amplification. Molecular-based taxonomy has also enabled the development of techniques for the study of environmental DNA (eDNA). Previous studies indicated the 16S mitochondrial ribosomal RNA (rRNA) gene as a promising target for this application; however, 16S rRNA sequences are available for only a limited number of mosquito species. In addition, although primers for the 16S rRNA gene were designed years ago, they are based on limited numbers of mosquito sequences. Thus, the aims of this study were to: (i) design pan-mosquito 16S rRNA gene primers; (ii) using these primers, generate a 16S rRNA gene mosquito reference library (with a focus on mosquitoes present in Italy); and (iii) compare the discriminatory power of the 16S rRNA gene with two widely used molecular markers, cytochrome* c* oxidase subunit 1 mitochondrial gene (COI) and internal transcribed spacer 2 (ITS2).

**Methods:**

A total of six mosquito genera (28 mosquito species) were included in this study: *Aedes* (*n* = 16 species), *Anopheles* (5 species), *Coquillettidia* (1 species), *Culex* (3 species), *Culiseta* (2 species) and *Uranotaenia* (1 species). DNA was extracted from the whole mosquito body, and more than one specimen for each species was included in the analysis. Sanger sequencing was used to generate DNA sequences that were then analysed through the Barcode of Life Data Systems (BOLD). Phylogenetic analyses were also performed.

**Results:**

Novel 16S rDNA gene, COI and ITS2 sequences were generated. The 16S rRNA gene was shown to possess sufficient informativeness for the identification of mosquito species, with a discriminatory power equivalent to that of COI.

**Conclusions:**

This study contributes to the generation of DNA barcode libraries, focussed on Italian mosquitoes, with a significant increase in the number of 16S rRNA gene sequences. We hope that these novel sequences will provide a resource for studies on the biodiversity, monitoring and metabarcoding of mosquitoes, including eDNA-based approaches.

**Graphical abstract:**

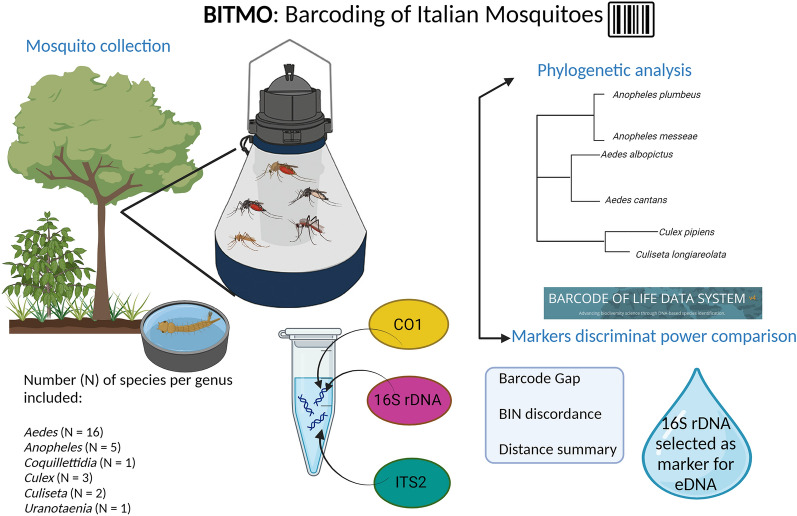

**Supplementary Information:**

The online version contains supplementary material available at 10.1186/s13071-024-06478-0.

## Background

Mosquitoes (Diptera: Culicidae) are recognised as the most impactful arthropod disease vectors worldwide. They inhabit diverse habitats, ranging from the tropics to the Arctic Circle. The taxonomic diversity of Culicidae is extensive: they are divided into two subfamilies (Anophelinae and Culicinae), with a total of 112 genera and 3718 species officially recognised as of June 2023 [1, 2]. Of these, 88 species are known as vectors of human pathogens, with an additional 243 species suspected to be potential vectors [3]

The Italian mosquito fauna is composed of 65 mosquito species belonging to eight genera from both subfamilies, including the invasive and now established tiger mosquito *Aedes albopictus;* of these, 65 species, 34 are competent to transmit human and animal pathogens [4]. This list of Italian mosquitoes also includes species that have not been detected in Italy in the last 50 years, among which is *Aedes aegypti,* whose last recording dates back to 1971. In recent years, the number of Italian mosquitoes has increased due to the introduction of alien species characterised by invasive capability, mostly originating from Asia. In addition to the already well-established *Ae. albopictus*, *Aedes koreicus* and *Aedes japonicus japonicus* are currently spreading quickly across the Italian Peninsula [5, 6].

Invasive mosquito species (IMS) represent a threat to public health because they can act as vectors for a variety of disease agents that are normally restricted to other geographical areas, in particular the tropical regions [7, 8]. For example, autochthonous cases of chikungunya and dengue fevers have recently been described in southern, central and northern areas of Italy in 2007, 2017, 2020 and 2023 [9–13].

Accurate entomological monitoring is pivotal for the early detection and control of IMS [14]. Traditional monitoring methods are based on the morphological identification of mosquito species at different life stages, using dichotomous keys [7, 15, 16]. These methods are associated with a number of disadvantages: they are time-consuming, require a high level of taxonomic expertise and present issues in the case of damaged samples or life stages whose morphology is poorly differentiated [1, 15]. Moreover, certain mosquito species that have distinct vectorial capacities can appear morphologically indistinguishable [16], making the development of alternative monitoring strategies urgent. Approaches based on molecular markers could offer a helpful alternative for morphological identifications of adult and immature stages. These strategies require less time from the operator, especially in the case of large sets of specimens that may be processed in parallel, do not need a high level of expertise and allow identification at all the life stages or of damaged samples. Furthermore, they discriminate between cryptic species or species belonging to complexes [15, 16]. In recent years, high-throughput sequencing has been applied in DNA barcoding, allowing the concurrent identification of multiple species from single environmental samples. This approach is known as metabarcoding [17]. Until now the biggest sequencing effort for mosquitoes has been focussed on the mitochondrial cytochrome *c* oxidase I (COI) gene, but other markers have also been proposed for mosquito barcoding [1]: the mitochondrial gene coding for 16S ribosomal RNA (rRNA; hereafter 16S rDNA) and the nuclear marker internal transcribed spacer 2 (ITS2).

ITS2 is a non-coding region that forms a spacer region between the 5.8S and 28S rRNA genes. It is a highly variable region and thus a useful marker to reveal even minor genetic discontinuities. However, several studies [16, 18, 19], including one on Italian cryptic species [20], suggested that this marker might not be optimal to identify mosquito species. Indeed, the insect nuclear genome usually contains a high number of rRNA gene copies, repeated in tandem, with ITS2 presenting intra-individual variation between the different copies of this spacer. Furthermore, it frequently presents high levels of inter-individual polymorphisms. Thus, it may be infeasible/impractical to employ this marker for the identification of single individuals [1, 21].

The COI gene is a widely used marker for animal identification and is considered a sort of gold standard in DNA barcoding for several reasons: (i) it is conserved in almost all eukaryotic organisms; (ii) it is present in a high copy number (being associated with mitochondria); and (iii) it has a higher substitution rate compared to the nuclear genes, allowing a higher level of intra- and inter-specific discrimination [22]. COI is maternally inherited in mosquitoes, with almost no recombination [1]. Barcoding studies have been conducted in mosquitoes by several authors and these have generally been based on COI; these investigations have been performed in different countries, including India, Belgium, Pakistan, Portugal, Sri Lanka, French Guiana, China, England, Canada, Mexico, Croatia, Estonia and Thailand [16, 23–35]. Notably, a comprehensive barcoding study on mosquito COI is still lacking in Italy. In most of the quoted studies, results based on morphological observation and COI barcoding were highly congruent.

In 2024, Maurício Moraes Zenker and colleagues assessed the availability of mosquito reference sequences for COI and ITS2 and found low coverage for both markers (28.4–30.11% for COI in the Barcode of Life Data Systems [BOLD] + GenBank, and 12.32% for ITS2 in GenBank), with countries hosting the higher biodiversity having the lower reference sequence coverage [36].

Despite COI being regarded as a standard in barcoding applications [37, 38], doubts have been expressed on whether it is the best marker in all possible contexts [39]. The main issue is that possible primer binding sites in this gene are not well conserved, especially due to synonymous substitutions, possibly leading to biases in amplification and, thus, in under- or over-representation of taxa in bulk samples. Moreover, variations at less constrained sites become saturated between distantly related taxa as a result of homoplasy, [40, 41]. DNA barcoding and metabarcoding have important applications in environmental DNA (eDNA) studies. eDNA-based investigations rely on detectable DNA traces that organisms release in the environment and on their persistence, which allows for the detection of rare or elusive species [42]. COI has widely been used in eDNA studies, even though this marker is affected by the limits reported above, and by the length of PCR amplicons [1]. To try to overcome this problem, short but still informative molecular markers have been investigated in the last 20 years [42, 43]. The design of primers targeting markers with these characteristics can be challenging, to the point that some authors referred to this goal as the “search for the Holy Grail” [44, 45].

Another mitochondrial marker, 16S rDNA, has been shown to be effective in insect barcoding and metabarcoding [44, 46, 47]. 16S rDNA evolves slower than COI and is characterised by conserved sequence stretches flanked by highly variable regions due to its stem-loop structures [48]. As a result, barcoding studies targeting this region often provide broader taxonomic coverage than markers based on COI [44, 46]. However, when all available insect reference databases are considered, there are threefold more reference sequences for the COI marker than for 16S rDNA [46]. Thus, 16S rDNA can be regarded as a good candidate to be developed as a barcode for mosquito identification and eDNA studies. In all cases, the identification and testing of primers appropriate for DNA metabarcoding requires the availability of exhaustive reference databases, over which the taxonomic coverage and resolution of potential primers can be tested [44, 49–52]. It is thus necessary to build a more complete reference library for mosquitoes for this mitochondrial gene. The primary aim of this study was thus to generate a reference barcode 16S rDNA library for Italian mosquitoes. In addition, to determine the actual efficacy of this marker in mosquito barcoding, we also generated libraries for COI and ITS2, starting from the same individuals, and compared the discriminatory power of the three markers.

## Methods

### Mosquito collection and morphological identification

A total of 28 mosquito species were included in this study. Mosquitoes were collected as larvae and adults by various collaborators during a monitoring activity conducted between 2021 and 2023 in Italy. The collected mosquitoes were raised under standard laboratory conditions [53], and at the pupal stage they were transferred into mosquito cages to facilitate adult-stage emergence. Field-collected adults were captured using CDC-CO_2_ traps or BG Sentinel traps (Biogents AG, Regensburg, Germany).

All mosquito adults were morphologically identified using previously described morphological features under a stereomicroscope (model IC90 E; Leica, Wetzlar, Germany) [54–57]. When possible, more than one specimen for each species was included in the analysis. The complete list of mosquito species and the numbers of individuals collected for this study are summarised in Table 1. After morphological identification, all of the specimens were individually placed in a 1.5-ml tube with 70% ethanol and stored at − 20 °C until DNA extraction and amplification.

**Table 1:** Specific details on the mosquito samples used in this study

Mosquito species	Samples (n)	Barcoding index number (BIN)	Collection site in Italy	Latitude	Longitude
*Aedes aegypti*	3	BIN: AEI9358	Insectary at the UniMI	45.4766	9.2335
*Aedes albopictus*	2	BIN: AAA5870	Bergamo (Trescore Balneario)	45.7173	9.8438
*Aedes cantans*	1	BIN: AAB1098	Venezia (Campagna Lupia)	45.3612	12.1390
*Aedes caspius*	3	BIN: AAB7911:	Venezia (Chioggia)	45.1989	12.2837
*Aedes cinereus*	2	BIN: AAP8897	Bologna (Baricella, Molinella)	44.6760, 44.5693	11.5659, 11.6561
*Aedes communis*	2	BIN: AAA6148	Bergamo (Trescore Balneario)	45.7173	9.8438
*Aedes detritus*	2	BIN: AAM2826	Ferrara (Ostellato, Comacchio)	44.7428, 44.7944	11.9539, 12.2576
*Aedes geniculatus*	2	BIN: ADZ3180, AEG2154	Bergamo (Parzanica) + Trento (Terragnolo)	45.7363, 45.8833	10.0339 11.1500
*Aedes japonicus*	4	BIN: AAC5210	Como (Garzola)	45.8095	9.1042
*Aedes koreicus*	2	BIN: ACB6413	Como (Tavernerio)	45.8090	9.1276
*Aedes mariae*	3	BIN: AED2194:	Latina (San Felice Circeo)	41.2218	13.0683
*Aedes pulcritarsis*^a^	2	BIN: AAN1645	Bologna	44.4626	11.3203
*Aedes rusticus*	2	BIN: AAM5033	Modena (Formigine)	44.6139	10.7921
*Aedes sticticus*	1	BIN: ACB9122	Pordenone (Cordenons)	45.9835	12.6742
*Aedes vexans*	3	BIN: AAA7067	Rovigo (Occhiobello)	44.8905	11.6042
*Aedes zammitii*	3	BIN: AAB7911	Bari (Polignano a Mare)	40.9853	17.2486
*Anopheles labranchiae*	1	BIN: ABY8238	IZSVe	42.7127	10.9854
*Anopheles maculipennis*	2	BIN: AAA9632	Brescia (Porle) + Verona (Isola della Scala)	45.6076, 45.2905	10.3781, 11.0184
*Anopheles messeae*^b^	2	BIN: ABY8239	Verona (Isola della Scala)	45.2905	11.0184
*Anopheles petragnani*	4	BIN: AAA9648	Como (Garzola)	45.8095	9.1042
*Anopheles plumbeus*	4	BIN: AAN3326	Biella (Pollone)	45.5805	7.9952
*Coquillettidia richiardii*	2	BIN: AAS0072	Mantova (Monzambano)	45.3691	10.6427
*Culex hortensis*	3	BIN: AAI5767	Brescia (Caino)	45.6131	10.3254
*Culex mimeticus*	2	BIN: AAM3149	Bologna (Valsamoggia)	44.4801	11.0838
*Culex pipiens*	5	BIN: AAA4751	Bergamo (Foresto Sparso)	45.6953	9.9000
*Culiseta annulata*	5	BIN: AAD6954	Bologna (Sasso Marconi)	44.3484	11.2936
*Culiseta longiareolata*	3	BIN: AAP0901	Como (Civiglio)	45.8103	9.1137
*Uranotaenia unguiculata*	2	BIN: ADJ6199	Bologna (Pianoro)	44.4132	11.3408

*UniMI* University of Milan

^a^See the study of Harbach and Wilkerson [58]

^b^See the study of Calzolari and colleagues [20]

### Mitochondrial 16S rDNA primers design

Novel primers targeting the mosquito mitochondrial 16S rDNA were designed using available 16S rDNA sequences or complete mitochondrial genomes of mosquitoes present in GenBank as of October 2022 (*n* = 967) (Additional file 3: Table S3). The sequences were aligned together using MUSCLE, a multiple sequence alignment method, and the alignment was visualised using the ARB software package to identify the most conserved regions for manual primer design. As a result, five possible primers were designed: two forward and three reverse (Additional file 4: Figure S1). All six of these combinations were tested in silico with the online Multiple Primer Analyzer tool by Thermo Fisher Scientific (https://www.thermofisher.com/it/en/home/brands/thermo-scientific/molecular-biology/molecular-biology-learning-center/molecular-biology-resource-library/thermo-scientific-web-tools/multiple-primer-analyzer.html) and in vitro PCR on known mosquito samples (data not shown).

The selected pair of primers (Culic_m16S_F91 and Culic_m16S_R555) was preferred due to the longer product and thus higher resolution, while an alternative pair (Culic_m16S_F91 and Culic_m16S_R503) of primers was applied in few cases when amplification with the first combination was unsuccessful.

### DNA extraction and PCR amplification

DNA was extracted from the whole mosquito body using the DNeasy Blood and Tissue Kit (Qiagen, Valencia, CA, USA), following the manufacturer’s protocol with only minor adjustments. After DNA extraction, three different markers were amplified for each sample: COI, ITS2 and 16S rDNA (see Table 2 for specific information). For the amplification of COI, the conventional primers LCO1490/HCO2198 were used [59], and the PCR cycling parameters were: a first denaturation step of 94 °C for 5 min, followed by 40 cycles of 30 s at 94 °C, 45 s at 49 °C 45 s and 1 min at 72 °C, with a final extension step of 72 °C 10 min.

**Table 2:** Existing and newly designed primers used to amplify three target gene regions with the amplification size

Target	Forward sequence	Reverse sequence	Size (bp)	Source
COI	LCO1490 5′-GGTCAACAAATCATAAAGATATTGG-3'	HCO2198 5′-TAAACTTCAGGGGTGACCAAAAATCA-3'	648	Folmer et al.[59]
ITS2	5.8S 5′-TGTGAACTGCAGACGACATG-3’	28S 5′-ATGCTTAAATTGGGGGGTA-3’	Approx. 370	Collins and Paskewitz [60]
16S rDNA	Culic_m16S_F91 5′-TAGAAACCAACCTGGCTTAC-3’	Culic_m16S_R555 5’-GTGCGAAGGTAGCATAATCA-3’ Culic_m16S_R503 5’-ATGGTTGAATGAGATATATACTGT-3’	Approx. 464 Approx. 412	This study

*COI* Cytochrome *c* oxidase I,* ITS2* internal transcribed spacer 2,* rDNA* ribosomal DNA

For 16S rDNA, the following PCR cycling parameters were applied: An initial denaturation for 5 min at 95 °C, followed by 35 cycles of 30 s at 95 °C, 30 s at 54 °C and 1 min at 72°, with a final extension step of 5 min at 72 °C.

The ITS2 nuclear marker was amplified using the 5.8S and 28S primers using the protocol suggested by Collins and Paskewitz [60] with some minimal adaptations.

After checking the quality of the amplifications by electrophoresis in agarose gels, PCR products were sent to Eurofins Genomics to be sequenced through Sanger-based technology (Eurofins Genomics, Ebersberg, Germany).

### Phylogenetic analyses

The obtained sequences were checked for quality and edited using the DNA sequencing software Chromas (Technelysium Pty Ltd, Brisbane, Australia). For phylogenetic analyses we analysed the dataset for each marker (COI, 16S rDNA and ITS2) separately. In order to confirm our species identification, we added at least one published sequence per species to the COI dataset. For this purpose, whenever present, Italian sequences deposited in the BOLD were preferred; in the absence of Italian sequences, the preferred order of sequences was sequences from BOLD from other European countries, sequences from BOLD from any location and finally sequences in NCBI GenBank with the same order of geographical preference. BOLD was preferred over GenBank due to the higher taxonomic curation of the former. Such an approach allowed us to obtain a reliable and comprehensive comparative framework as reference for all of the following analyses. Specifically, the COI phylogeny allowed us to “anchor” all analyses on the same specimens with the other markers, in particular the 16S rDNA, for which reference sequences are limited or completely lacking for most analysed species in both GeneBank and BOLD databases.

For each dataset, all sequences were aligned using the multiple sequence alignment method MUSCLE [61] and trimmed using Gblocks [62] integrated in the Seaview 5.0 software application [63] with default settings. The best substitution model for each dataset was determined using jModelTest 2.1.7 [64]. Then, maximum likelihood phylogenetic trees were inferred with phyML [65] using 100 bootstrap replicates. Considering that multiple studies (e.g. [66]) consistently reported monophyly of both Culicinae and Anophelinae, and thus the separation of these two subfamilies, we rooted our trees on the branch separating the representatives of the two families (i.e. between genus *Anopheles* and the others).

### Molecular species delimitation

All of the newly obtained sequences were uploaded in the BOLD database. The COI sequences were automatically assigned Barcode Index Numbers (BINs) using the REfin Single Linkage clustering (RESL) approach based on operational taxonomic units (OTUs) [35, 67]. A ‘BIN discordance analysis’ was carried out to verify the concordance between such BIN assignments and morphological taxonomic designation.

Molecular species delimitation analyses were performed on each of the newly obtained COI and 16S rDNA sequence datasets using the tools available in the BOLD workbench. The ITS2 was excluded from such analyses due to its insufficient informativeness (see Results).

The tools employed included ‘Distance Summary’ and 'Barcode Gap Analysis’. The former was applied to investigate the sequence divergence among barcode sequences at the conspecific, congeneric and confamilial levels. The latter, i.e. Barcode Gap Analysis, was meant to assess the distance to the nearest neighbour for each of the species considered. For these analyses, all sequences shorter than 100 bp were discarded, as well as, in the respective analysis, singletons, i.e. species represented by a single sequence or genera represented by a single species.

The Pairwise Kimura distances were calculated after aligning with the MUSCLE aligner and applying pairwise deletion of ambiguous bases/gaps. Then, following Madeira et al., a ‘distance summary’ was obtained for the distances within species, genus and the whole Culicidae family [16]. Moreover, a ‘Barcode Gap Analysis’ was performed by comparing the distance of each sequence to its furthest conspecific (the maximum intra-specific genetic distance) and to its nearest non-conspecific (the minimum interspecific genetic distance) [68, 69].

### Data availability

**Fig. 1:**
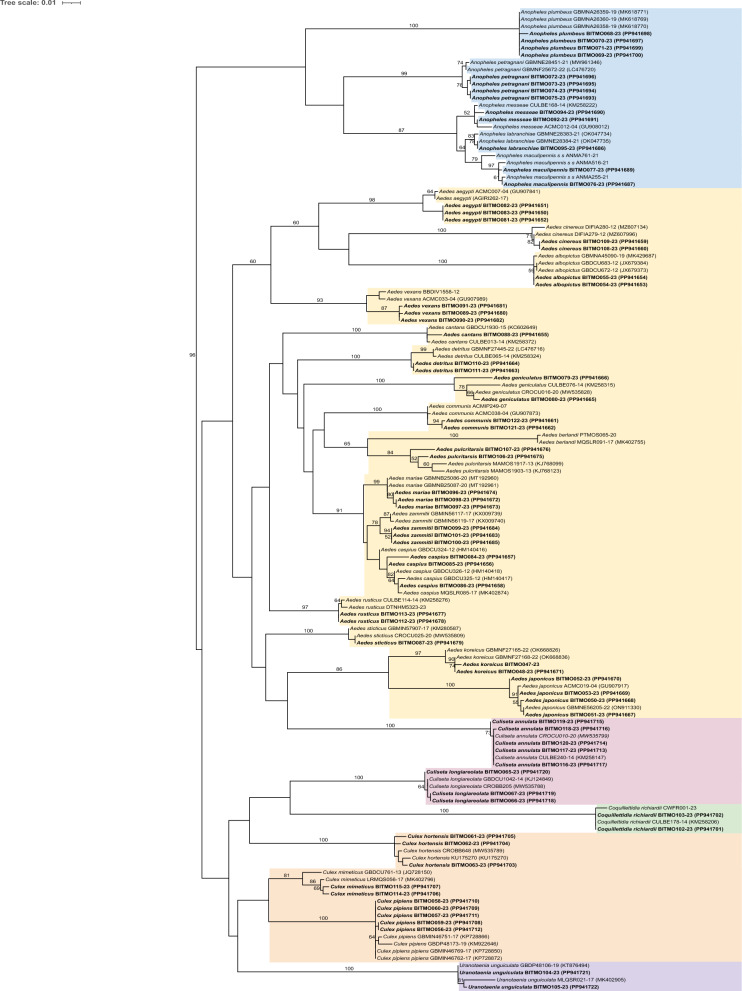
Maximum likelihood phylogenetic tree with the GTR+I+G model of the COI sequences of 28 Italian mosquito species, including newly sequenced ones (shown in bold font) and the sequences downloaded from BOLD and GenBank (regular font). Sequence names are accompanied by the respective BOLD identifier and NCBI accession number (in brackets). Bootstrap values (with 100 replicates) > 50 are reported on the branches. The different mosquito genera are indicated by different colours: *Anopheles*, (blue), *Aedes* (yellow), *Culiseta* (pink), *Coquilletidia* (green), *Culex* (orange), *Uranotaenia* (purple). BOLD Barcode of Life Data Systems; COI, cytochrome* c* oxidase 1; NCBI, National Center for Biotechnology Information

All newly obtained sequences were deposited into BOLD [68] into a novel dedicated project named BITMO (Barcoding of ITalian MOsquitoes), as well as into the NCBI GenBank (accession numbers: COI, PP941650—PP941722; 16s rDNA, PP835598—PP835668; ITS2, PP835545—PP835597). Accession numbers to each individual sequence, for all the examined individual mosquitoes, are reported at the terminal nodes of the trees presented in Figs. 1 and 3, and in Additional file 5: Figure S2. No datasets were generated or analysed during the current study.

## Results

After the mosquitoes had been identified and the DNA extraction from the samples, sequences were obtained for a total of 74 individuals representing 28 mosquito species present in Italy, belonging to six genera: *Aedes* (16 species), *Anopheles* (5), *Coquillettidia* (1), *Culex* (3), *Culiseta* (2) and *Uranotaenia* (1). Most of the species were represented by at least two individuals; for three species (*Aedes sticticus*, *Aedes cantans* and *Anopheles labranchiae*), only single individuals were examined. The results obtained for each marker are presented separately in the following sections.

### Cytochrome* c* oxidase 1 gene

A successful amplification of the COI gene from all 74 mosquito specimens was obtained for the 28 included species. The length of the obtained sequences ranged from 464 to 686 bp, with the overwhelming majority of sequences (68/74) being longer than 600 bp. The large-scale COI phylogeny of Culicidae was consistent with expectations, with a highly supported separation between the subfamilies Culicinae and Anophelinae (Fig. 1). In contrast, deep evolutionary relationships among and within genera of Culicinae were poorly supported and although sequences from congeneric species largely clustered together, not all genera are monophyletic for this gene. In particular, species from the genera *Culiseta*,* Aedes* and *Culex* had been placed into polyphyletic or paraphyletic clusters. However, phylogenetically misplaced species generally had poor bootstrap support in the reconstructed COI-based positioning (< 50). The limits of COI for the reconstruction of the overall mosquito phylogeny have already been reported [30].

All species, including the sequences available in the databases and the newly obtained ones (i.e. 74 + 65 available sequences), formed monophyletic clusters, mostly with high support values (21/28 with > 80 bootstrap support). Therefore, the COI tree confirmed morphological assignment, at least for the newly generated sequences. As indicated by branch lengths, some species show some intra-specific variability, which may be attributed to the different geographic origin of the different samples, in particular when sequences retrieved from the databases and the newly obtained ones are considered, such as in the cases of *Aedes vexans* and *Ae. aegypti*.

From the 28 species, the automatic BOLD assignment identified 28 BINs among the novel 74 sequences. This result was largely consistent with the morphological identification, with two exceptions. In one case, BIN discordance consisted in the clustering of the two sister species *Aedes zammitii* and *Aedes caspius* as a single BIN (AAB7911). In the other case, individuals of *Aedes geniculatus* were assigned to two different BINs (ADZ3180; AEG2154).

For the ‘Distance summary’ and “Barcode Gap Analysis,” we used 72 sequences out of the 74 novel sequences. Two specimens for which the 16S rDNA sequences had not been obtained (see below) were discarded in order to have fully comparable datasets.

**Fig. 2:**
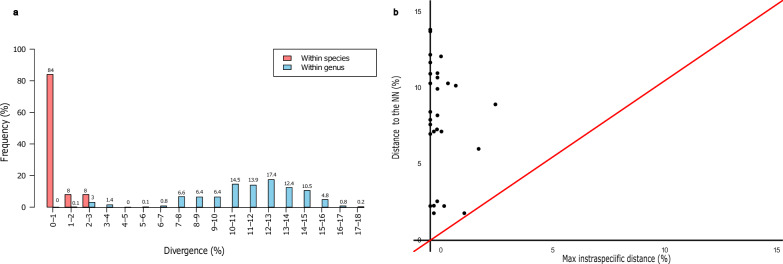
**a** Distance summary analysis for COI, showing the within-species normalised distribution for the divergence within species (red) and within genus (blue). **b** Scatterplot showing the maximum intra-specific distances vs the inter-specific distance for each species in the COI. The red diagonal line represents the equal values for the two distances, as a threshold for adequate species delimitation (barcoding gap). COI, Cytochrome* c* oxidase 1

As expected, mean genetic distance sharply increased from lower to higher taxonomic levels, being 0.30% within species, 11.30%, between species within genera and 14.56% between species belonging to different genera within the same families (Table 3). However, distance ranges within genera and families were quite broad, resulting in partial overlaps of the within-genus range with the within-species and within-family ranges (Table 3). Nevertheless, the distribution of distances within different groups was overall quite distinct, with the overlaps being due to a small number of “outlier” distances (Fig. 2a).

**Table 3:** Summary of genetic distance for the cytochrome* c* oxidase 1 gene in mosquitoes at different taxonomic levels

Label	Number	Taxa (*n*)	Comparisons (*n*)	Minimum distance (%)	Mean distance (%)	Maximum distance (%)	Standard error distance (%)
Within species	69	25	72	0.00	0.30	2.91	0.01
Within genus	68	4	747	1.78	11.30	17.10	0.00
Within family	72	1	1737	10.14	14.56	20.41	0.00

The taxonomic levels are ranked from lower to higher, i.e. species to genus to family

The “Barcode Gap Analysis” indicated a perfect separation at the species level, since for all species the distance to the closest non-conspecific (i.e. nearest neighbour [NN]) was higher than the respective maximum intra-specific distance (Fig. 2b). Specifically, 26 of the 28 species showed a low intra-specific diversity (< 2%), with the two exceptions being *Aedes pulcritarsis* (2.16%) and *Ae. geniculatus* (2.91%) (Additional file 1: Table S1), consistent with the relatively longer branch lengths (Fig. 1). In addition, the three singletons (8 species) showed no variability (intra-specific divergence = 0%). Furthermore, all interspecific distances to the respective NNs were always > 2%, except between the closely related *Ae. caspius* and *Ae. zammitii* (1.78%)*.*

### 16S rDNA mitochondrial gene

A total of 72 sequences, derived from the included 28 mosquito species, were obtained for the 16S rDNA mitochondrial marker, with at least one specimen for each investigated species. The length of the sequences obtained ranged from 247 to 438 bp, with the vast majority (66/72) being longer than 350 bp. Due to the almost complete lack of reference sequences in Genbank and BOLD, only the newly obtained sequences were considered for the phylogenetic analysis. The reliability of species identification was guaranteed by our accurate morphological observation and the use of COI as an additional marker for species identification.

**Fig. 3:**
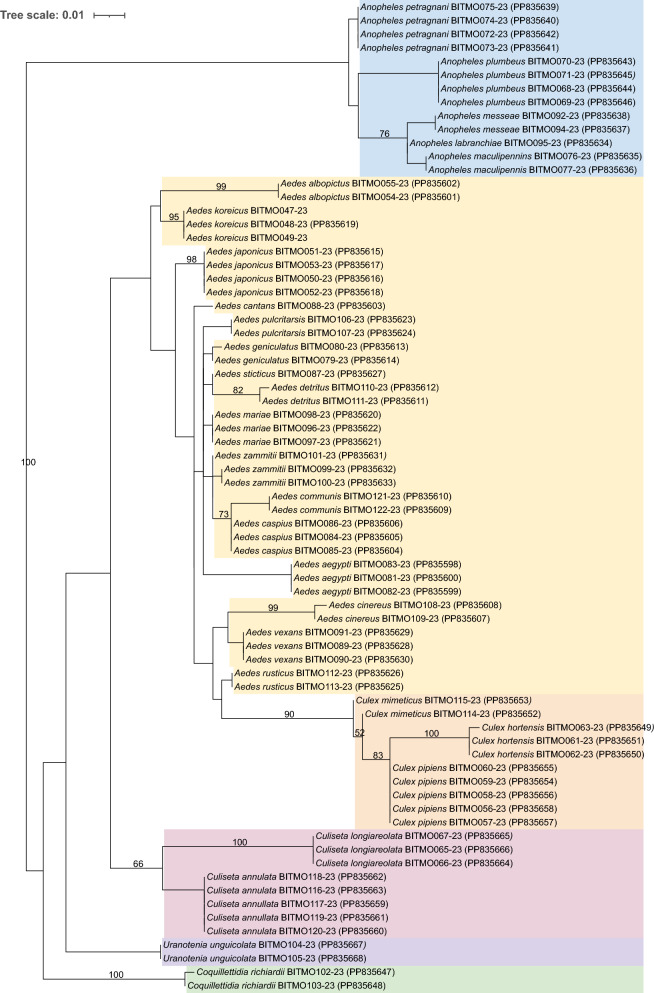
Maximum likelihood phylogenetic tree with the GTR+I+G model of the newly obtained 16S rDNA sequences from the 28 Italian mosquito species. Sequence names are accompanied by the respective BOLD identifier and NCBI accession number (in brackets). Bootstrap values (with 100 replicates) > 50 are reported on the branches. The different mosquito genera are indicated by different colours: *Anopheles*, (blue), *Aedes* (yellow), *Culiseta* (pink), *Coquilletidia* (green), *Culex* (orange), *Uranotaenia* (purple). BOLD, Barcode of Life Data Systems; COI, cytochrome* c* oxidase 1; NCBI, National Center for Biotechnology Information

The 16S rDNA-based phylogeny was overall consistent with the one based on COI (Fig. 3). Specifically, there was a sharp and fully supported separation between Anophelinae and Culicinae. Interestingly, the consistency of molecular phylogeny with the taxonomic assignment at the species level was even higher than that observed in the COI tree. Indeed, the only non-monophyletic genus was *Aedes*, and also in this case the nodes that would indicate its paraphyly were poorly supported (< 50). Conversely, phylogenetic relationships at the species level were slightly less resolved as compared with the COI phylogeny. Indeed, 26 of the 28 species were monophyletic (only 8 with > 80 support), with the exceptions being *Ae. zammitii* and *Culex mimeticus*, although in both of these cases the non-monophyly was poorly supported (< 50).

As with the COI analysis, the mean genetic distances increased from lower to higher taxonomic levels, but at lower values than those observed for the COI analysis, being 0.03% within species, 2.66% within genera, and 8.33% within families (Table 4). In contrast to the COI analyses, there was no overlap between the distance ranges within species (maximum 0.35%) and within genera (minimum 0.51%), while there was still an overlap, though smaller, of the distance ranges between genera (maximum 6.50%) and families (minimum 3.56%).

**Table 4:** Summary of genetic distance for 16S ribosomal DNA gene in mosquitoes at different taxonomic levels

Label	Number	Taxa (*n*)	Comparisons (*n*)	Minimum distance (%)	Mean distance (%)	Maximum distance (%)	Standard error distance (%)
Within species	69	25	72	0.00	0.03	0.35	0.00
Within genus	68	4	747	0.51	2.66	6.50	0.00
Within family	72	1	1737	3.56	8.33	15.78	0.00

The taxonomic levels are ranked from lower to higher, i.e. species to genus to family

**Fig. 4:**
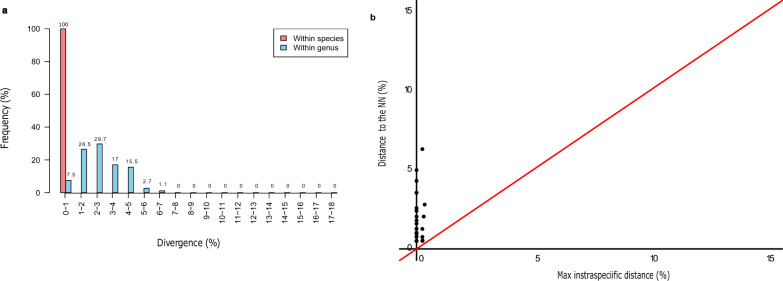
**a** Distance summary analysis for the 16S rDNA, showing the within-species normalised distribution for the divergence within species (red) and within genus (blue). **b** The scatterplot showing the maximum intra-specific distances vs the inter-specific distance for each species in the 16S rDNA. The red diagonal line represents the equal values for the two distances, as a threshold for adequate species delimitation (barcoding gap). rDNA, Ribosomal DNA

Consistent with the complete separation of the distances within species and genera, the results obtained from the “Barcode Gap Analysis” confirmed the presence of a barcoding gap (Fig. 4a), thereby supporting the capability of the 16S rDNA marker to effectively distinguish between species. The intra-specific diversity was much lower than that observed for COI, being at most 0.35% for *Aedes cinereus*. The minimum inter-specific distances to the respective NNs were smaller as well, ranging from 0.51% (6 cases) to 6.32% of *Coquilletidia richiardii* with respect to *Culex mimeticus.* Nevertheless, none of such distances to NNs outweighed the corresponding maximum intra-specific distance (Additional file 2: Table S2).

### Internal transcribed spacer 2 marker

The amplification and sequencing of the ITS2 was successful for 65 individuals out of the 74 examined. In particular, ITS2 could not be amplified and sequenced for the following specimens: *Ae. koreicus* (BITMO001_02), *Ae. japonicus* (BITMO002_02; BITMO002_03), *Ae. geniculatus* (BITMO010_01), *Ae. zammitii* (BITMO019_02), *Uranotaenia unguiculata* (BITMO021; BITMO021_01), *Aedes pulcritarsis* (BITMO022_01) and *Culiseta annulata* (BITMO027_01). We obtained sequences of at least one specimen for the 28 investigated species, the missing one being *Ur. unguiculata*. The obtained sequences ranged from 104 to 401 bp, with approximately half of the sequences (*n* = 28) being longer than 300 bp.

For consistency with the analyses on COI and 16S rDNA, the two specimens for which no 16S rDNA was obtained were excluded from the phylogeny analysis on ITS2. The obtained phylogenetic tree was partially consistent with those previously inferred on the two mitochondrial markers (Additional file 5: Figure S2). However, the results were unsatisfactory in several respects. In particular, the topology of the ITS2-based tree was poorly supported for several nodes and was partially inconsistent with that of the trees based on the other markers. For example, the separation between Culicinae and Anophelinae had relatively low support (< 80), and the *Culex* and *Aedes* genera were non-monophyletic, with the former split into seven different branches. Moreover, five of the 27 included species were non-monophyletic, including *Culex mimeticus* BITMO026_01, which branched within a clade of sequences affiliated to the *Aedes* genus as sister group of *Aedes rusticus*, with high support (> 80).

This apparent lower quality of the phylogeny is likely due to insufficient phylogenetic signal, as a consequence of the short length of the sequences obtained and of the intrinsic properties of this marker. This result is consistent with previous studies, which already evidenced that, due to multiple divergent ITS2 copies present in the same individual, it is frequently impractical to obtain long and high-quality sequences with PCR followed by direct Sanger sequencing only [1].

Based on the short length and deemed insufficient informativeness of the obtained sequences, no further analyses on sequence divergence for species discrimination were performed on this marker.

## Discussion

In this study, we aimed to generate and validate reference libraries for DNA barcoding for native and alien species of Culicidae. We designed pan-mosquito 16S rRNA gene primers and we used these set of primers to generate a 16S rRNA gene mosquito reference library focussed on mosquito species present in southern Europe; finally, we successfully compared the discriminatory power of the 16S rRNA gene with two commonly used molecular markers, COI and ITS2. In summary, novel sequences for these three markers were produced, with the 16S rRNA gene demonstrating sufficient informativeness to identify mosquito species, with a discriminatory power comparable to that of the COI gene.

### Cytochrome* c* oxidase 1 and 16S rDNA mitochondrial genes

To verify the suitability of the selected COI primers to effectively distinguish mosquito species (see Results), we investigated the presence of the Barcoding Gap, defined as the difference between the highest intra-specific and the lowest inter-specific distance in congeneric species [16, 70, 71]. Analyses were run directly on the BOLD database; these included only our new sequenced samples, listed in a dedicated project named BITMO (Barcoding of Italian Mosquitoes). Previous investigations on DNA barcoding on mosquitoes were mainly based on this marker (i.e. COI). In the present study, in general, molecular and morphological characterisations were shown to be highly consistent in these studies [72]. We found high consistency between morphology and COI in relation with species identification. However, COI-based DNA barcoding yielded results that have not always been regarded as reliable in Diptera. It is still unclear whether the observed inconsistencies were caused by the lack of phylogenetic signal in the COI gene, particularly at deep taxonomic level, by the methods used for phylogenetic reconstruction or the non-monophyly of investigated taxa due to mitochondrial DNA introgression or incomplete lineage sorting [73]. A further potential issue of COI is the length of the metabarcodes, as they often are > 600 bp, which hampers inexpensive sequencing technologies (e.g. Illumina 2×300; Illumina Inc., San Diego, CA, USA).

Once the efficacy of COI had been assessed in terms of species identification and barcoding gap, the analyses were run on the 16S rDNA sequence obtained from the same samples, with the results confirming that also this marker is suitable for species identification in mosquitoes. For this purpose, more 16S rDNA sequences from other mosquito species, both from Italy and from other geographic locations, would be useful.

Our current study represents the first comprehensive investigation of 16S rDNA sequence diversity in the mosquito fauna of a specific region. Previous to the present study, only a few studies had investigated 16S rDNA in mosquito populations, and these usually included a limited number of species [74–78].

Comparing results obtained on COI and 16S rDNA, we would summarise and comment our results as follows: (i) the barcoding gap in 16S rDNA is less pronounced than in COI, possible due to the lower rate of evolution of the 16S rDNA gene; (ii) the 16S rDNA marker proved suitable for mosquito identification in our setting, despite this less-pronounced gap; (iii) the expected amplified DNA size from the designed 16S rDNA-based marker is shorter than that of the marker based on COI gene (464 bp for 16S; 648 bp for COI). This is extremely valuable for eDNA studies, where shorter DNA fragments are expected to be present in the samples due to DNA degradation [38, 70]. We emphasise that previous studies, which focused on different taxa, showed that 16S rDNA is also a good candidate for eDNA studies [44, 47, 79–81].

### Internal transcribed spacer 2 marker

Our analysis of the ITS2 marker did not lead to the expected results in terms of its suitability for species differentiation in mosquitoes. Our results are in agreement with those of previous studies that reported the limits of this marker, particularly in relation with standard Sanger-based sequencing. Batovska and colleagues [82] investigated the ITS2 marker in mosquitos, analysing 88 sequences obtained with Sanger sequencing; only 18 of the 26 species considered were resolved monophyletically. In comparison, 24 species were detected with Illumina sequencing. These authors concluded that ITS2 Sanger sequencing is not completely satisfactory for mosquitoes species identification when dealing with numerous species [82]. Due to the pronounced intra-specific variability, the ITS marker could be very useful in delimitating species in mosquito complexes, but, for this characteristic, alignment of distantly related species is often difficult.

## Conclusions

In conclusion, this study contributes to the building of national reference libraries for mosquito species present in Italy, targeting three markers: COI, ITS2 and 16S rDNA. COI was confirmed to be an effective barcode for mosquitoes and suitable for species identification. In our experimental settings, ITS2 was associated with a few limitations, likely due to its nature as a multicopy DNA sequence, which entails intra-individual variability. 16S rDNA has proved to be suitable tool for mosquito identification, with results comparable to those based on COI. The fact that the use of shorter amplicons is possible when using this marker as compared with COI make 16S rDNA worthy of further investigation for its application in eDNA-based monitoring. Therefore, in consideration of our results, we would propose that this marker is assessed for its efficacy also in studies on the presence of mosquito DNA in the environment.

## Supplementary Information

**Additional file 1: Table S1.** Details for species comparison for the COI marker; the mean and maximum intra-specific values are compared to the nearest neighbour for each species. When the species is a singleton, the intra-specific values are represented as N/A

**Additional file 2: Table S2. **Details for species comparison for the 16S marker; the mean and maximum intra-specific values are compared to the nearest neighbour for each species. When the species is a singleton, the intra-specific values are represented as N/A.

**Additional file 3: Table S3.** Available 16S rDNA sequences or complete mitochondrial genomes of mosquitoes present in GenBank as of October 2022 used for the design of new primers.

**Additional file 4: Figure S1.** The five primers targeting the mitochondrial 16S rRNA gene of mosquitoes, presented relative to the mitochondrial genome of *Aedes albopictus* strain Rimini.

**Additional file 5: Figure S2.** Maximum likelihood phylogenetic tree with the GTR+I+G model of the newly obtained ITS2 sequences of 28 Italian mosquito species. Sequence names are accompanied by the respective BOLD identifier and NCBI accession number. Bootstrap valuesabove 50% are reported on the branches. Different colours indicate the mosquito genera: *Anopheles *, *Aedes *, *Culiseta *, *Coquilletidia *, *Culex *, *Uranotaenia *.

## Data Availability

The datasets generated in this study were deposited in the NCBI with the accession numbers: COI, PP941650—PP941722; 16 s rDNA, PP835598—PP835668; ITS2, PP835545—PP835597) and in BOLD with the sequence page numbers from BITMO047-23 to BITMO122-23.

## Declarations

Ethics approval and consent to participate.

Not applicable.
