# Supplementary material for: Barcoding of Italian mosquitoes (BITMO): generation and validation of DNA barcoding reference libraries for native and alien species of Culicidae
Source: Parasit Vectors. 2024 Sep 28;17:407. doi: 10.1186/s13071-024-06478-0 (PMC11439297; doi:10.1186/s13071-024-06478-0)
Supplement: Supplementary file 1 — Additional file 1: Table S1. Details for species comparison for the COI marker; the mean and maximum intra-specific values are compared to the nearest neighbour for each species. When the species is a singleton, the intra-specific values are represented as N/A [file 13071_2024_6478_MOESM1_ESM.docx]

| Order | Family | Species | Mean Intra-Sp | Max Intra-Sp | Nearest Species | Nearest Neighbour | Distance to NN |
| --- | --- | --- | --- | --- | --- | --- | --- |
| Diptera | Culicidae | *Aedes aegypti* | 0 | 0 | *Aedes vexans* | BITMO091-23 | 10.93 |
| Diptera | Culicidae | *Aedes albopictus* | 0 | 0 | *Aedes aegypti* | BITMO083-23 | 12.18 |
| Diptera | Culicidae | *Aedes berlandi* | 2.16 | 2.16 | *Aedes zammitti* | BITMO100-23 | 6 |
| Diptera | Culicidae | *Aedes cantans* | N/A | 0 | *Aedes caspius* | BITMO085-23 | 8.43 |
| Diptera | Culicidae | *Aedes caspius* | 1.05 | 1.52 | *Aedes zammitti* | BITMO066-23 | 1.78 |
| Diptera | Culicidae | *Aedes cinereus* | 0 | 0 | *Aedes rusticus* | BITMO112-23 | 11.67 |
| Diptera | Culicidae | *Aedes communis* | 0.3 | 0.3 | *Aedes koreicus* | BITMO096-23 | 7.28 |
| Diptera | Culicidae | *Aedes detritus* | 0 | 0 | *Aedes zammitti* | BITMO100-23 | 6.98 |
| Diptera | Culicidae | *Aedes geniculatus* | 2.91 | 2.91 | *Aedes rusticus* | BITMO112-23 | 8.92 |
| Diptera | Culicidae | *Aedes japonicus* | 0.5 | 0.79 | *Aedes koreicus* | BITMO047-23 | 10.3 |
| Diptera | Culicidae | *Aedes koreicus* | 0 | 0 | *Aedes japonicus* | BITMO052-23 | 10.3 |
| Diptera | Culicidae | *Aedes mariae* | 0.1 | 0.16 | *Aedes caspius* | BITMO085-23 | 2.27 |
| Diptera | Culicidae | *Aedes rusticus* | 0 | 0 | *Aedes zammitti* | BITMO100-23 | 7.6 |
| Diptera | Culicidae | *Aedes sticticus* | N/A | 0 | *Aedes mariae* | BITMO097-23 | 7.91 |
| Diptera | Culicidae | *Aedes vexans* | 0.21 | 0.32 | *Aedes rusticus* | BITMO113-23 | 8.2 |
| Diptera | Culicidae | *Aedes zammitti* | 0.05 | 0.16 | *Aedes caspius* | BITMO085-23 | 1.78 |
| Diptera | Culicidae | *Anopheles labranchiae* | N/A | 0 | *Anopheles messeae* | BITMO092-23 | 2.25 |
| Diptera | Culicidae | *Anopheles maculipennis* | 0.31 | 0.31 | *Anopheles messeae* | BITMO092-23 | 2.56 |
| Diptera | Culicidae | *Anopheles messeae* | 0.62 | 0.62 | *Anopheles labranchiae* | BITMO095-23 | 2.25 |
| Diptera | Culicidae | *Anopheles petragnani* | 0.21 | 0.32 | *Anopheles messeae* | BITMO092-23 | 9.94 |
| Diptera | Culicidae | *Anopheles plumbeus* | 0 | 0 | *Anopheles labranchiae* | BITMO095-23 | 13.71 |
| Diptera | Culicidae | *Coquillettidia richiardii* | 0 | 0 | *Culex mimeticus* | BITMO114-23 | 13.42 |
| Diptera | Culicidae | *Culex hortensis* | 1.08 | 1.14 | *Culex pipiens* | BITMO060-23 | 10.15 |
| Diptera | Culicidae | *Culex mimeticus* | 0.47 | 0.47 | *Culex pipiens* | BITMO060-23 | 7.23 |
| Diptera | Culicidae | *Culex pipiens* | 0.1 | 0.16 | *Culex mimeticus* | BITMO114-23 | 7.23 |
| Diptera | Culicidae | *Culiseta annulata* | 0.13 | 0.32 | *Aedes rusticus* | BITMO112-23 | 10.68 |
| Diptera | Culicidae | *Culiseta longiareolata* | 0.21 | 0.32 | *Aedes rusticus* | BITMO112-23 | 10.97 |
| Diptera | Culicidae | *Uranotaenia unguiculata* | 0.48 | 0.48 | *Culex mimeticus* | BITMO114-23 | 12.23 |
