# Supplementary material for: Barcoding of Italian mosquitoes (BITMO): generation and validation of DNA barcoding reference libraries for native and alien species of Culicidae
Source: Parasit Vectors. 2024 Sep 28;17:407. doi: 10.1186/s13071-024-06478-0 (PMC11439297; doi:10.1186/s13071-024-06478-0)
Supplement: Supplementary file 2 — Additional file 2: Table S2. Details for species comparison for the 16S marker; the mean and maximum intra-specific values are compared to the nearest neighbour for each species. When the species is a singleton, the intra-specific values are represented as N/A. [file 13071_2024_6478_MOESM2_ESM.docx]

| Order | Family | Species | Mean Intra-Sp | Max Intra-Sp | Nearest Species | Nearest Neighbour | Distance to NN |
| --- | --- | --- | --- | --- | --- | --- | --- |
| Diptera | Culicidae | *Aedes aegypti* | 0 | 0 | *Aedes zammitii* | BITMO101-23 | 2.41 |
| Diptera | Culicidae | *Aedes albopictus* | 0 | 0 | *Aedes koreicus* | BITMO048-23 | 3.56 |
| Diptera | Culicidae | *Aedes berlandi* | 0 | 0 | *Aedes geniculatus* | BITMO079-23 | 1.01 |
| Diptera | Culicidae | *Aedes cantans* | N/A | 0 | *Aedes geniculatus* | BITMO079-23 | 1.03 |
| Diptera | Culicidae | *Aedes caspius* | 0 | 0 | *Aedes mariae* | BITMO098-23 | 0.51 |
| Diptera | Culicidae | *Aedes cinereus* | 0.35 | 0.35 | *Aedes vexans* | BITMO089-23 | 2.81 |
| Diptera | Culicidae | *Aedes communis* | 0 | 0 | *Aedes caspius* | BITMO085-23 | 0.97 |
| Diptera | Culicidae | *Aedes detritus* | 0.25 | 0.25 | *Aedes sticticus* | BITMO087-23 | 1.27 |
| Diptera | Culicidae | *Aedes geniculatus* | 0.25 | 0.25 | *Aedes sticticus* | BITMO087-23 | 0.51 |
| Diptera | Culicidae | *Aedes japonicus* | 0 | 0 | *Aedes zammitii* | BITMO101-23 | 1.6 |
| Diptera | Culicidae | *Aedes koreicus* | 0 | 0 | *Aedes rusticus* | BITMO112-23 | 2.05 |
| Diptera | Culicidae | *Aedes mariae* | 0 | 0 | *Aedes caspius* | BITMO085-23 | 0.51 |
| Diptera | Culicidae | *Aedes rusticus* | 0 | 0 | *Aedes vexans* | BITMO090-23 | 1.28 |
| Diptera | Culicidae | *Aedes sticticus* | N/A | 0 | *Aedes geniculatus* | BITMO079-23 | 0.51 |
| Diptera | Culicidae | *Aedes vexans* | 0 | 0 | *Aedes rusticus* | BITMO112-23 | 1.28 |
| Diptera | Culicidae | *Aedes zammitti* | 0.18 | 0.26 | *Aedes sticticus* | BITMO087-23 | 0.53 |
| Diptera | Culicidae | *Anopheles labranchiae* | N/A | 0 | *Anopheles*  *maculipennis* | BITMO076-23 | 0.51 |
| Diptera | Culicidae | *Anopheles maculipennis* | 0 | 0 | *Anopheles*  *labranchiae* | BITMO095-23 | 0.51 |
| Diptera | Culicidae | *Anopheles messeae* | 0 | 0 | *Anopheles*  *labranchiae* | BITMO095-23 | 0.77 |
| Diptera | Culicidae | *Anopheles petragnani* | 0 | 0 | *Anopheles*  *labranchiae* | BITMO095-23 | 1.81 |
| Diptera | Culicidae | *Anopheles plumbeus* | 0 | 0 | *Anopheles*  *petragnani* | BITMO072-23 | 2.59 |
| Diptera | Culicidae | *Coquillettidia richiardii* | 0.25 | 0.25 | *Culex mimeticus* | BITMO114-23 | 6.32 |
| Diptera | Culicidae | *Culex hortensis* | 0.1 | 0.31 | *Culex pipiens* | BITMO058-23 | 2.05 |
| Diptera | Culicidae | *Culex mimeticus* | 0.25 | 0.25 | *Culex pipiens* | BITMO056-23 | 0.76 |
| Diptera | Culicidae | *Culex pipiens* | 0 | 0 | *Culex mimeticus* | BITMO114-23 | 0.76 |
| Diptera | Culicidae | *Culiseta annulata* | 0 | 0 | *Aedes koreicus* | BITMO048-23 | 3.56 |
| Diptera | Culicidae | *Culiseta longiareolata* | 0 | 0 | *Culiseta annulata* | BITMO120-23 | 4.31 |
| Diptera | Culicidae | *Uranotaenia unguiculata* | 0 | 0 | *Aedes koreicus* | BITMO047-23 | 4.99 |
