# Supplementary figures and images for: Barcoding of Italian mosquitoes (BITMO): generation and validation of DNA barcoding reference libraries for native and alien species of Culicidae
Source: Parasit Vectors. 2024 Sep 28;17:407. doi: 10.1186/s13071-024-06478-0 (PMC11439297; doi:10.1186/s13071-024-06478-0)

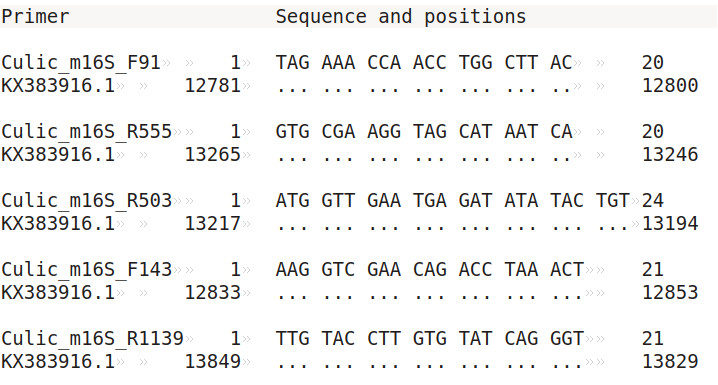

Supplement: Supplementary file 4 — Additional file 4: Figure S1. The five primers targeting the mitochondrial 16S rRNA gene of mosquitoes, presented relative to the mitochondrial genome of Aedes albopictus strain Rimini. [file 13071_2024_6478_MOESM4_ESM.jpg]

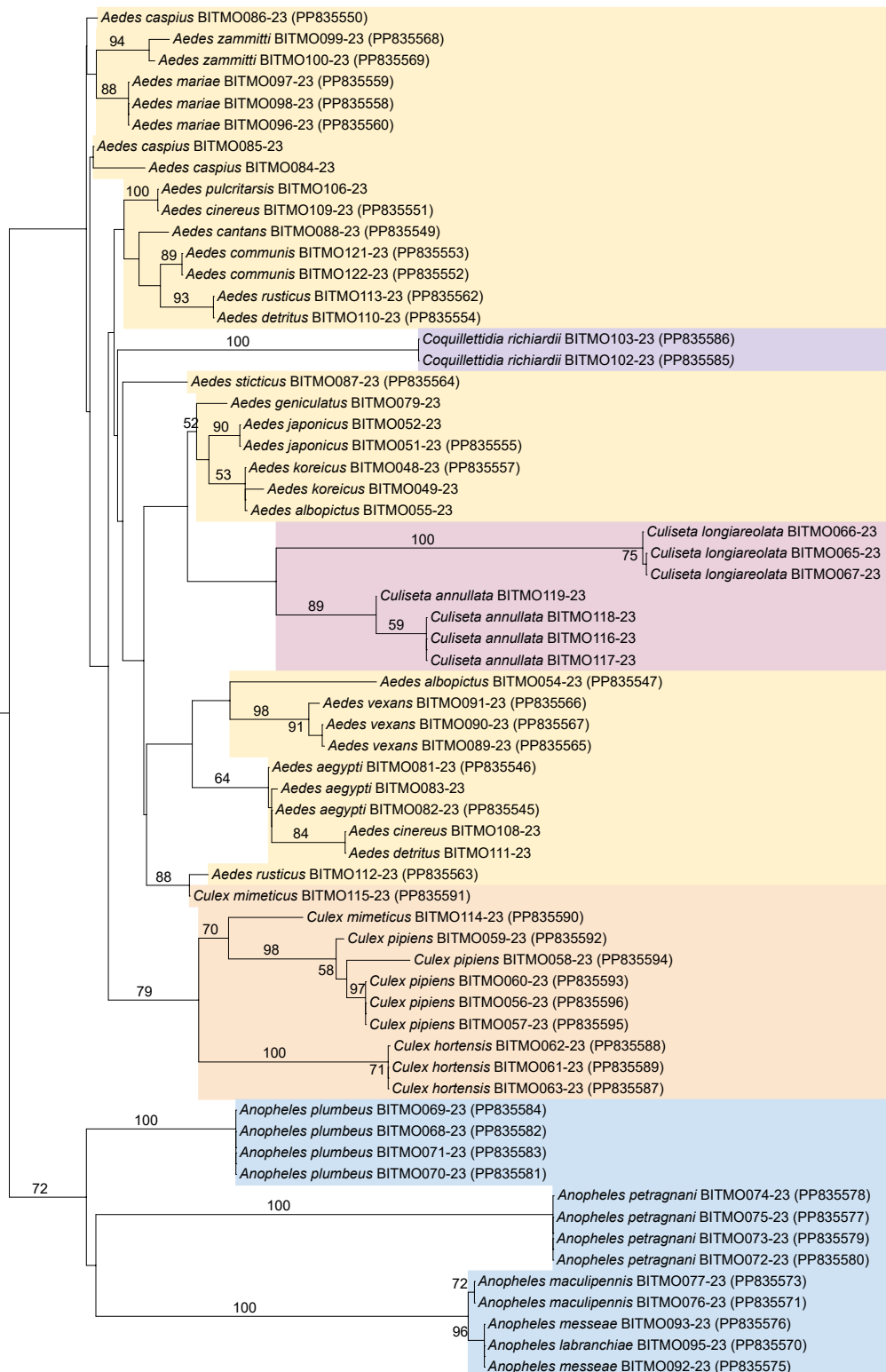

Supplement: Supplementary file 5 — Additional file 5: Figure S2. Maximum likelihood phylogenetic tree with the GTR+I+G model of the newly obtained ITS2 sequences of 28 Italian mosquito species. Sequence names are accompanied by the respective BOLD identifier and NCBI accession number. Bootstrap valuesabove 50% are reported on the branches. Different colours indicate the mosquito genera: Anopheles , Aedes , Culiseta , Coquilletidia , Culex , Uranotaenia . [file 13071_2024_6478_MOESM5_ESM.pdf]
